# Prebiotic galactooligosaccharides activate mucin and pectic galactan utilization pathways in the human gut symbiont *Bacteroides thetaiotaomicron*

**DOI:** 10.1038/srep40478

**Published:** 2017-01-16

**Authors:** Alicia Lammerts van Bueren, Marieke Mulder, Sander van Leeuwen, Lubbert Dijkhuizen

**Affiliations:** 1 Microbial Physiology, Groningen Biomolecular Sciences and Biotechnology Institute (GBB), University of Groningen, Groningen, The Netherlands

## Abstract

Galactooligosaccharides (GOS) are prebiotic carbohydrates that impart changes in the gut bacterial composition of formula-fed infants to more closely resemble that of breast-fed infants. Consuming human milk oligosaccharides (HMOs) provides specific bacterial strains with an advantage for colonizing the infant intestine. These same effects are seen in infants after GOS consumption, however GOS are very complex mixtures and the underlying molecular mechanisms of how GOS mimic HMOs are relatively unknown. Here we studied the effects of GOS utilization on a prominent gut symbiont, *Bacteroides thetaiotaomicron,* which has been previously shown to consume HMOs via mucin O-glycan degradation pathways. We show that several pathways for targeting O-mucin glycans are activated in *B. thetaiotaomicron* by GOS, as well as the galactan utilization sytem. Characterization of the endo-galactanase from this system identified activity on various longer GOS substrates while a subset of GOS compounds were identified as potential activators of mucin glycan metabolism in *B. thetaiotaomicron*. Our results show that GOS functions as an inducer of mucin-glycan pathways while providing a nutrient source in the form of β-(1 → 4)-galactan. These metabolic features of GOS mixtures may serve to explain the beneficial effects that are seen for GOS supplemented infant formula.

The human gastrointestinal tract is colonized by millions of bacteria that form a complex ecosystem between the food transiting the gut and epithelial lining of the intestinal tract^1^. Bacterial species are adapted to the dynamic conditions of the gastrointestinal tract where they must acquire nutrients from a variety of sources that are quickly transiting through the intestinal lumen^2^. Gut bacteria have evolved extensive metabolic systems to harvest nutrients they may encounter in the gut. The food we consume therefore dictates to a large extent what bacterial species are present in our gut microbiota^3^.

Glycans as dietary fibres are consumed by specialized gut bacteria^4^,^5^,^6^. In the absence of dietary glycans, the mucosal layer serves as a carbohydrate-rich alternative nutritional source for gut microbiota^7^,^8^. As such, microbial composition is also dependent on mucins^9^. Mucin is composed of high molecular weight glycoproteins that form a thick gel-like layer on the surface of the gut epithelium which acts as lubrication and a protective barrier between the intestinal lumen^10^. Within mucus, core-glycans are covalently linked to serine or threonine to form complex O-linked glycan structures^11^. The type of O-glycans produced in the human intestinal tract is dependent upon many factors encoded within the human genome, including expression of genes responsible for mucin synthesis (MUC), ones secretor type and/or blood group type (such as Lewis/ABO, etc)^12^. Bacteria that able to degrade mucin glycans are more easily adaptable to the changing intestinal environment and have an advantage in colonizing the mucosal surface for establishing themselves as a core species in the gastrointestinal (GI) tract^7^,^11^.

The bacterium *Bacteroides thetaiotaomicron* is a prominent member of the human microbiota and resides in the distal gut. It is characterized by its complex catabolic systems built up of carbohydrate-active enzymes and transporters that function to degrade a wide range of complex host and dietary polysaccharides into their individual monosaccharide components^13^,^14^. These catabolic systems are arranged in defined operons called polysaccharide utilization loci (PULs) that make up approximately 20% of the *B. thetaiotaomicron* genome and target specific polysaccharides for degradation^4^,^5^. *B. thetaiotaomicron* is found to contain PULs that target dietary polysaccharides, microbial polysaccharides, and human glycans such as mucins. The expression of PULs targeting mucin glycans was demonstrated to be important in colonization, persistence, and mother-to-infant transmission of *B. thetaiotaomicron*^8^. Human milk oligosaccharides (HMOs) found in mothers milk facilitated mother-to-infant transmission by inducing the expression of a distinct subset of mucin O-glycan utilization PULs^15^, suggesting that HMOs can facilitate bacterial transmission to infants by activating mucin glycan utilization pathways.

In humans, mother’s natural milk contains approximately 8% (10 g/L) HMOs of which there are approximately 200 structures that have been shown to have beneficial impacts on infant health^16^,^17^. These include facilitating bacterial colonization^18^ and protection from pathogens^19^,^20^. Galactooligosaccharides (GOS) are non-digestible carbohydrates that have been shown to elicit similar beneficial health effects as HMOs^21^. Infants fed formulas supplemented with GOS develop a gut microbiome that more closely resembles the microbiomes of breast-fed infants, when compared to those fed formula without GOS^22^,^23^.

GOS are synthesized on a large scale by incubating specific β-galactosidase enzymes with high concentrations of lactose where the enzymes form complex GOS mixtures via transglycosylation reactions that contain many molecules of differing length, linkage type and branching^24^,^25^,^26^,^27^. When consumed, GOS reach the distal colon similar to HMOs where they are degraded by resident bacteria^28^, promoting the growth of bacterial families, including *Bifidobacteriaceae* and *Bacteroidaceae*, in the intestine of infants in a similar ratio to that found in the gut microbiota of breast-fed infants. GOS are very complex mixtures^25^, and very little is known about what components within GOS offer the same HMOs-like effects.

In this investigation we used *B. thetaiotaomicron* as a model bacterium to identify the potential effects of GOS on infant gut associated bacterial species. We identified two distinct mechanisms directed at GOS metabolism by *B. thetaiotaomicron*: firstly, extended linear β-(1 → 4)-linked GOS molecules were degraded via the action of an extracellular GH53 endo-β-(1 → 4)-galactanase that is encoded within a galactan utilization PUL. Secondly, a subset of PULs directed at O-glycan and host glycan metabolism were activated in *B. thetaiotaomicron* also previously implicated in metabolism of HMOs. We deduced that these PULs may be activated due to the presence of a subset of GOS compounds, namely branched GOS and lactose-disaccharide derivatives. Together, we show that specific GOS molecules mimic HMOs by expressing enzymes and transport components from PULs indicated in mucin-O-glycan degradation in *B. thetaiotaomicron* while a specific endo-β-galactanase directed at dietary fibre catabolism plays an important role in GOS degradation. This is the first study to identify the molecular details of GOS utilization by *B. thetaiotaomicron* from which we can speculate how specific GOS compounds may elicit responses similar to HMOs. These results provide a basis for making more selective prebiotic preparations for infant formulas and for broader prebiotic and dietary fibre applications.

## Results

### *B. thetaiotaomicron* consumes distinct components of GOS mixtures

**Figure 1:**
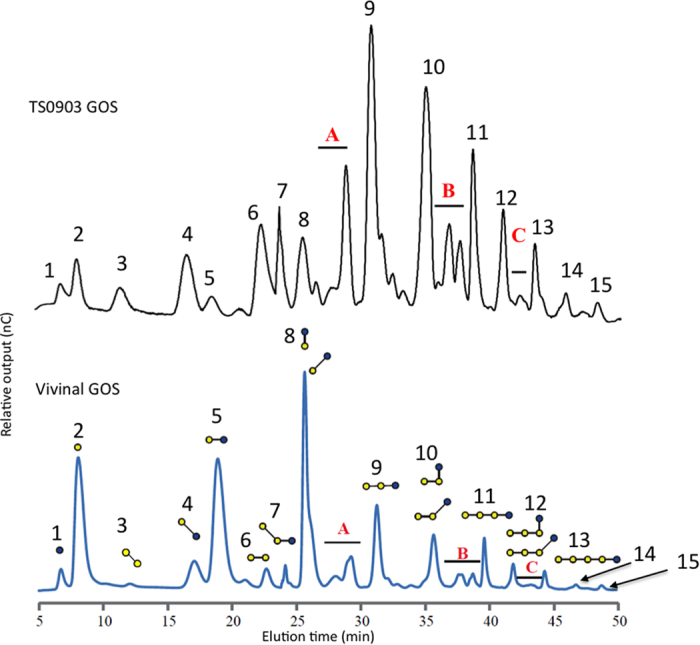
GOS profile analysis of Vivinal GOS (bottom) and purified GOS (top). Compounds were separated by HPAEC-PAD and identified based on previously published information^29^. Galactose is depicted as yellow circles, glucose is depicted as blue circles. **1.** D-glucose, **2.** D-galactose, **3.** β-D-Galp-(1 → 6)-β-D-Gal, **4.** β-D-Galp-(1 → 6)-β-D-Glc, **5.** β-D-Galp-(1 → 4)-β-D-Glc (lactose), **6.** β-D-Galp-(1 → 4)-β-D-Gal, **7.** β-D-Galp-(1 → 6)-β-D-Galp-(1 → 4)-β-D-Glc, **8.** β-D-Galp-(1 → 2)-β-D-Glc, β-D-Galp-(1 → 3)-β-D-Glc, (**A**) DP3 Branched GOS (left to right) β-D-Galp-(1 → 4)-[β-D-Galp-(1 → 6)-]D-Glcp, β-D-Galp-(1 → 2)-[β-D-Galp-(1 → 4)-]D-Glcp and β-D-Galp-(1 → 2)-[β-D-Galp-(1 → 6)-]D-Glcp, β-D-Galp-(1 → 3)-[β-D-Galp-(1 → 6)-]D-Glcp. **9.** β-D-Galp-(1 → 4)-β-D-Galp-(1 → 4)-β-D-Glc, **10.** β-D-Galp-(1 → 4)-β-D-Galp-(1 → 2)-β-D-Glc, β-D-Galp-(1 → 4)-β-D-Galp-(1 → 3)-β-D-Glc, (**B**) compounds in A) with an additional β-D-Galp-(1 → 4)-linked residue on the non-reducing end on either galactose residue. **11.** β-D-Galp-(1 → 4)-β-D-Galp-(1 → 4)-β-D-Galp-(1 → 4)-β-D-Glc, **12.** β-D-Galp-(1 → 4)-β-D-Galp-(1 → 4)-β-D-Galp-(1 → 2)-β-D-Glc, β-D-Galp-(1 → 4)-β-D-Galp-(1 → 4)-β-D-Galp-(1 → 3)-β-D-Glc, **C)** compounds in A) with an additional β-D-Galp-(1 → 4)-linked residues present on the non-reducing end of both galactose residues. **13.** β-D-Galp-(1 → 4)-β-D-Galp-(1 → 4)-β-D-Galp-(1 → 4)-β-D-Galp-(1 → 4)-β-D-Glc, **14.** β-D-Galp-(1 → 4)-β-D-Galp-(1 → 4)-β-D-Galp-(1 → 4)-β-D-Galp-(1 → 2)-β-D-Glc, β-D-Galp-(1 → 4)-β-D-Galp-(1 → 4)-β-D-Galp-(1 → 4)-β-D-Galp-(1 → 3)-β-D-Glc, **15.** β-D-Galp-(1 → 4)-β-D-Galp-(1 → 4)-β-D-Galp-(1 → 4)-β-D-Galp-(1 → 4)-β-D-Galp-(1 → 4)-β-D-Glc.

For this study, we made use of a purified GOS mixture (TS0903, provided by FrieslandCampina) based upon Vivinal GOS where the lactose starting material and other mono- and disaccharides have been removed. In essence, purified GOS represents a mixture of compounds that are available to the gut microbiota after the human digestible components (lactose, galactose and glucose) have been absorbed in the gut. We first analyzed the compounds present in the purified GOS mixture using HPAEC-PAD and compared this to the composition of Vivinal GOS which has been previously reported^29^. We could identify the majority of compounds within this purified GOS mixture, in terms of its carbohydrate content, linkage type and degree of polymerization (Fig. 1).

The purified GOS mixture has considerably higher amounts of linear GOS with a degree of polymerization (DP) of three than Vivinal GOS (30% versus 15%) (Table 1), with 4-galactosyllactose being the major peak (Fig. 1). In Vivinal GOS, the DP2 content is significantly greater than in TS0903 GOS (27% versus 8%); Vivinal GOS contained much higher amounts of the starting material lactose than purified GOS (19% versus 1%). Also, purified GOS contained greater amounts of DP4 and higher GOS than Vivinal GOS (38% versus 11%), and a higher proportion of branched compounds (17% versus 7%). We chose to use the purified GOS mixture in our studies because it consisted of the presumable indigestible portion of GOS, i.e. only those compounds which would enter the lower gastrointestinal tract after being exposed to human digestive enzymes in the small intestine.

**Figure 2:**
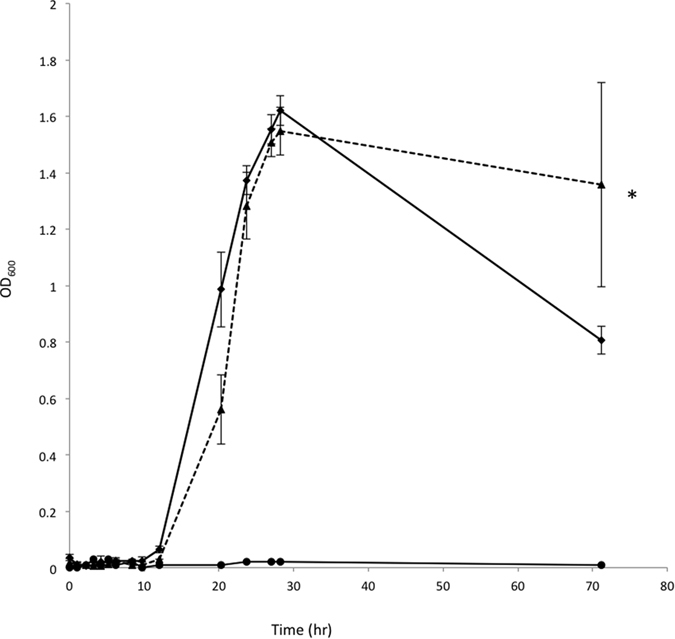
Growth on GOS mixture. Growth curves of *B. thetaiotaomicron* in a minimally defined medium with carbon sources added at a final concentration of 5 mg/ml. Hungate tubes flushed with gas containing 80%N_2_, 10%H_2_, 10% CO_2_. Readings were taken by placing Hungate tubes in the spectrophotometer at time intervals over a 4-day period. Glucose (diamonds), purified GOS (triangles) and carbon excluded control (circles). A non-parametric two-tailed Wilcoxon matched pairs signed rank test gives a P value = 0.0374 (P < 0.05, 95% confidence) statistically significant difference between glucose versus GOS (indicated by *).

**Figure 3:**
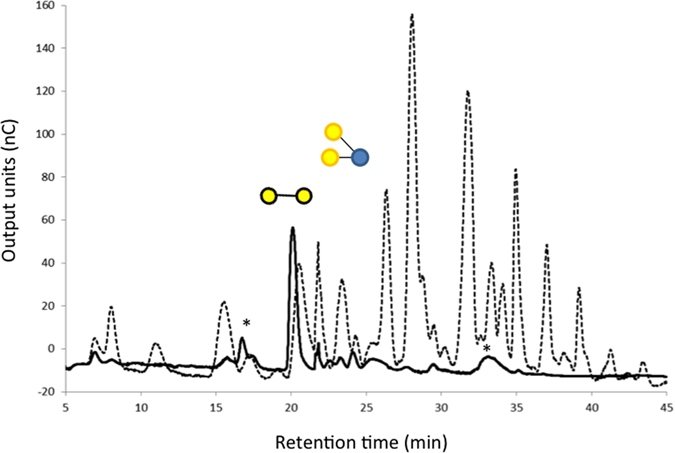
HPAEC-PAD separation and analysis of GOS mixture before (dotted line) and after growth (solid line) in *B. thetaiotaomicron* chemically defined minimal media plus GOS. Compounds identified in GOS mixture after growth: β-D-Galp-(1 → 4)-[β-D-Galp-(1 → 6)-]D-Glcp and galactobiose. Verification that the branched DP3 compound was β-D-Galp-(1 → 4)-[β-D-Galp-(1 → 6)-]D-Glcp was done by H^1^ NMR analysis (see Supplementary Material, Fig. 1).

To investigate metabolic properties of GOS consumption, we grew *B. thetaiotaomicron* in a carbon-limited minimal medium containing the purified GOS mixture at 5 mg/ml concentrations. *B. thetaiotaomicron* exhibited excellent growth on GOS (Fig. 2), reaching OD values greater than the control cultures containing an equal amount of glucose. A non-parametric two-tailed Wilcoxon matched pairs signed rank test resulted in a P value = 0.0374 (P < 0.05, 95% confidence) suggesting that over the 72 hours period of measured growth of *B. thetaiotaomicron* there is a statistically significant difference between GOS than glucose. Using HPAEC-PAD analysis of culture supernatants we confirmed that the majority of GOS compounds within the mixture were consumed by *B. thetaiotaomicron*. From a comparison of the HPAEC-PAD profiles of the starting mixture with the supernatant after bacterial growth, we identified the 2 compounds remaining in culture supernatants as a branched DP3 compound β-D-Galp-(1 → 4)-[β-D-Galp-(1 → 6)-]D-Glcp (see Supplementary Material, Fig. 1) and the disaccharide β-D-Galp-(1 → 4)-β-D-Gal which appears to be in a slightly larger quantity than the initial starting material (Fig. 3).

### Identification of galactan and mucin O-glycan PULS involved in *B. thetaiotaomicron* GOS consumption

In order to determine which polysaccharide utilization loci (PULs) within the *B. thetaiotaomicron* genome are induced to be expressed an produced as proteins in response to GOS, we took the culture supernatants after growth and analyzed for proteins and enzymes using mass spectrometry as has been previously described^30^. Previously it was found that *B. thetaiotaomicron* consumes hMOS using a distinct set of pathways attributed to mucin-O-glycan utilization^15^ (Table 2). Since GOS are thought to mimic properties of hMOS, we aimed to identify whether similar features were also activated by GOS in *B. thetaiotaomicron* (Table 2). As a confirmation that proteomics is a suitable method for detecting expressed components of PULs, we grew a culture of *B. thetaiotaomicron* on defined minimal medium containing 5 mg/ml purified HMOs substrates (Supplementary Material, Fig. 2) isolated from a donor milk sample as a sole carbon source to compare with previous HMOs qPCR gene expression analysis results^15^.

Within the sets of proteomics data we identified several SusC-like and SusD-like proteins from GOS and HMOs culture filtrates (Table 2, see Supplementary Material for full analytical report). SusC/SusD homologs^31^,^32^ form the major transport components of PULs and expression of these homologs is an indication of PUL activation by specific carbohydrate inducer substrates. We observed that TS0903 GOS molecules induce expression of several SusC and SusD homologs in *B. thetaiotaomicron* that are associated with hMOS and mucin utilization PULs (this study and ref. 15). In total, GOS induced the expression of five PUL SusC/D homologs that were also upregulated by HMOs and mucin^8^,^15^. These are BT_2032/BT_2033, BT_3958/BT3959, BT_4246/BT_4247, BT_4297/BT_4298 and BT_2805/BT_2806. In previous studies, BT_3958/BT_3959 was identified as a mucin O-glycan PUL upregulated by the Core 1 disaccharide, while BT_4246/BT_4247, BT_4297/BT_4298 and BT_2805/BT_2806 are upregulated in the presence of mucin O-glycans of undefined structure^8^,^15^. The PUL identified by BT_2032/BT_2033 is a separate PUL that appears to be induced by lactose and had not been previously identified as a PUL upregulated by mucin-O-glycans when compared to the galactose controls^15^. This specific PUL maybe expressed due to catabolite derepression caused by an accumulation of galactose; this PUL remains of undefined function.

In addition there were several individual SusC and SusD proteins identified in GOS culture supernatants that are associated with *B. thetaiotaomicron* HMOs consumption (Table 2). An additional SusC transporter (BT_0439) implicated in degradation of host glycans, and two additional SusD binding protein homologs induced by mucin O-glycans, BT_1039, and N-acetyllactosamine specific BT_2559, were identified.

**Figure 4:**
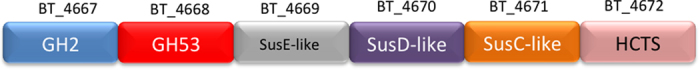
Graphical representation of the pectic galactan PUL from *B. thetaiotaomicron.* GH2, family 2 glycoside hydrolase, GH53, family 53 glycoside hydrolase, SusE, SUsD and SusC-like substrate binding and transport proteins, and hybrid two-component sensor regulator (HCTS). Protein identification numbers are listed above each PUL component.

SusC/D homologs of another prominent single PUL were identified in GOS culture supernatants that were neither found in hMOS nor in mucin culture supernatants. These were identified by the genetic ID loci BT_4670/BT_4671. These form the transport components of the putative galactan utilization system in *B. thetaiotaomicron*. Galactan utilization systems are employed by bacterial species for degradation and uptake of pectic galactan (Tabachnikov and Shoham, 2013)(Delangle *et al*., 2007), a linear polymer of β-(1 → 4)-linked galactose, that constitutes a structural component of pectin in plant cell walls. *B. thetaiotaomicron* encodes a PUL system for pectic galactan degradation (BT4667 – BT4673) which includes an encoded extracellular endo-β-(1 → 4)-galactanase (family GH53) for degrading larger galactan polymers (Fig. 4)^33^. Previous studies on GOS utilization by the probiotic bacterium *Bifidobacterium breve* have also shown that GH53 endo-β-galactanase activity is important for GOS utilization by Bifidobacteria^34^. Therefore we went on to investigate what the contribution of this endo-galactanase activity is towards utilization of GOS substrates.

### Influence of pectic galactan associated Endo-β-Galactanase

**Figure 5:**
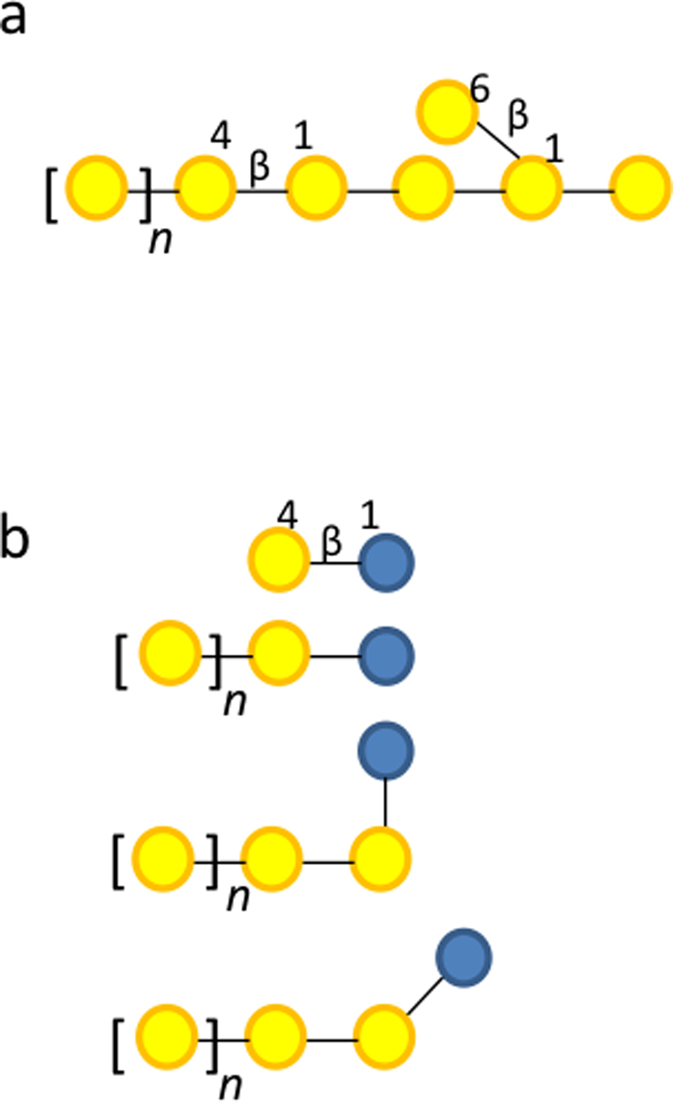
Graphical representation of the pectic galactan (**a**) and linear GOS (**b**) substrates showing the structural similarities between these two classes of compounds. Galactose is depicted as yellow circles, glucose is depicted as blue circles.

Comparison of GOS with pectic galactan reveals the same structural motifs in pectic galactan and in GOS compounds that have a higher DP and contain extensions of β-(1 → 4)-linked galactose residues (Fig. 5). Since pectic galactan is a dietary polysaccharide and has not been previously shown to be associated with HMOs effects (Table 2), galactan utilization PUL induction in *B. thetaiotaomicron* in the presence of GOS would be separate from mucin utilization – associated function. The proteome analysis from GOS culture supernatants identified the presence of the extracellular family GH53 endo-β-galactanase (BT4668) from the galactan utilization PUL while we did not find this enzyme in hMOS samples (Table 2) (Supplementary Material File 1). To study the potential effects of galactan PUL activation, we investigated the activity of the endo-β-galactanase GH53 enzyme (BT4668) with GOS substrates.

We cloned the gene BT4668 encoding endo-galactanase enzyme from *B. thetaiotaomicron* (herein called BtGH53), expressed it in *E. coli* and purified the enzyme to test for its activity on GOS. The enzyme was produced in soluble form and in large quantities (approx. 100 mg/L culture) and purified to >95% purity.

**Figure 6:**
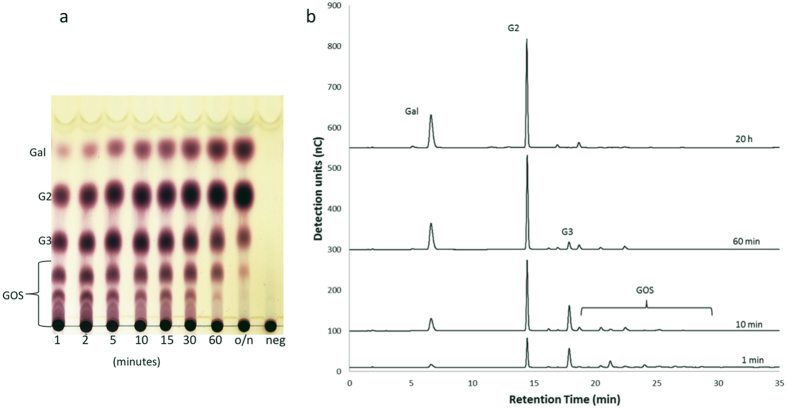
Enzymatic activity of BtGH53 on pectic galactan. (**a**) TLC analysis of products formed in time by BtGH53 incubated with potato galactan. (**b**) HPAEC-PAD profile of galactan derived products formed in time.

BtGH53 was found to be active on β-(1 → 4)-linked potato galactan (Fig. 6) but was not active on larch arabino galactan with β-(1 → 3)-linkage type (not shown). At the initial stages (1 min), BtGH53 converted potato galactan into GOS products with a degree of polymerization ranging from DP1-8 (Fig. 6). BtGH53 thus is an endo-acting β-galactanase enzyme. As the reaction progressed, GalDP4 and higher were hydrolyzed further to produce GalDP3, GalDP2 and galactose. At completion (circa 20 h) the main products were galactose (23%) and galactobiose (77%). These results suggest that BtGH53 is an endo-β-galactanase but also has some exo-activity in view of the presence of galactose and activity on GalDP3 substrate.

**Figure 7:**
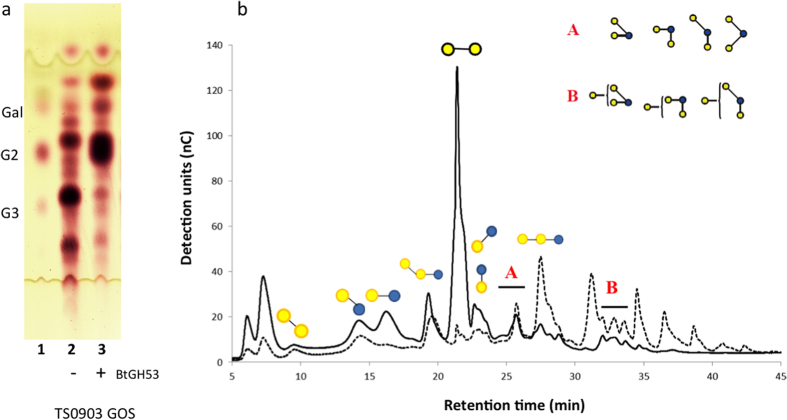
Enzymatic activity of BtGH53 on GOS. (**a**) TLC analysis of the products formed by enzymatic digestion of BtGH53 on GOS mixture. Lane 1: Pectic galactan products standard. Lane 2: GOS, Lane 3: GOS + BtGH53. 10 μl of BtGH53 was incubated with 10 μl of 1% GOS in reaction buffer for 16 h at 37 °C. Afterwards, 2 × 2 μl of each reaction was spotted on a TLC plate and run for 4 h in solvent system containing 3:1:1 isopropanol, ethyl acetate, water. Spots were visualized using stain containing 20% sulfuric acid, 80% methanol and 0.5% orcinol after heating at 110 °C for 20 min. (**b**) Corresponding HPAEC-PAD analysis of BtGH53 activity on GOS (dotted line: before incubation; solid line: after incubation) showing depletion of higher DP GOS molecules and accumulation of galactobiose. (A) DP3 Branched GOS (left to right) β-D-Galp-(1 → 4)-[β-D-Galp-(1 → 6)-]D-Glcp, β-D-Galp-(1 → 2)-[β-D-Galp-(1 → 4)-]D-Glcp and β-D-Galp-(1 → 2)-[β-D-Galp-(1 → 6)-]D-Glcp, β-D-Galp-(1 → 3)-[β-D-Galp-(1 → 6)-]D-Glcp. (B) compounds in (A) with an additional β-D-Galp-(1 → 4)-linked residue on the non-reducing end on either galactose residue.

Subsequently we tested for BtGH53 activity on the purified GOS mixture (Fig. 7). TLC analysis showed that BtGH53 facilitates hydrolysis of GOS that have a higher degree of polymerization forming galactobiose as the major product (Fig. 7a). By further examining the products of depolymerization using HPAEC-PAD we observed that BtGH53 hydrolyzes linear DP3 and higher GOS compounds (i.e. those compounds that contain at least two β-(1 → 4)-linked galactose residues) forming galactobiose as the main product (Fig. 7b). As an endo-β-galactanase BtGH53 thus acts on the linear high DP GOS compounds that share similar motifs to native pectic galactans (Fig. 5).

Also the branched DP4 GOS in region B were partially hydrolyzed, suggesting that GH53 may be cleaving terminal galactose from these compounds. GH53 enzymes from fungal origin have been observed to have exo-activity^35^, while the GH53 from *Bacillus licheniformis* did not^33^. Because we observed that BtGH53 degrades galactan DP3 products to DP2 plus galactose (Fig. 6), the exo-activity of BtGH53 on these substrates is plausible, however more experimental evidence, such as three-dimensional structural information of BtGH53 in complex with GOS substrate and enzymatic activity on individually purified GOS compounds would be beneficial to support this hypothesis.

We observed that the branched DP3 compounds β-D-Galp-(1 → 4)-[β-D-Galp-(1 → 6)-]D-Glcp and β-D-Galp-(1 → 2)-[β-D-Galp-(1 → 6)-]D-Glcp, β-D-Galp-(1 → 3)-[β-D-Galp-(1 → 6)-]D-Glcp and β-D-Galp-(1 → 4)-[β-D-Galp-(1 → 2)-]D-Glcp (region A) and the linear DP3 compound 6-galactosyl-lactose were recalcitrant to BtGH53 activity. The DP2 compounds Gal-β-(1 → 2)-Glc, Gal-β-(1 → 3)-Glc, Galβ-(1 → 6)-Gal, Galβ-(1 → 6)-Glc (allolactose) also were not cleaved. Additionally, we observed an accumulation of lactose and Gal-β-(1 → 2)-Glc/Gal-β-(1 → 3)-Glc after BtGH53 activity, likely arising from the hydrolysis of higher DP compounds (Fig. 7b).

**Figure 8:**
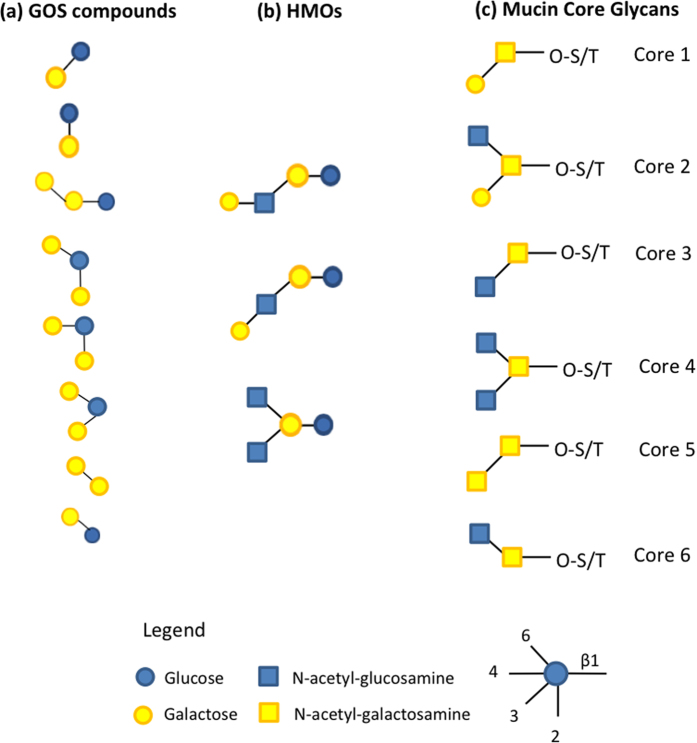
A schematic comparison of GOS compounds potentially inducing O-glycan utilization pathways (**a**) (this study) with core HMOs glycan structures (**b**) ref. 15 and mucin core glycan structures (**c**) ref. 11.

BtGH53 thus facilitates the degradation of linear and elongated branched GOS to produce galactobiose as the major product. The remaining GOS molecules that are not targeted by BtGH53 are DP2 Galβ-(1 → 2)-Glc and Gal β-(1 → 3)-Glc, Gal-β-(1 → 6)-Glc (allolactose) and Gal-β-(1 → 6)-Gal, linear DP3 6-GalLac, and the branched DP3 compounds β-D-Galp-(1 → 2)-[β-D-Galp-(1 → 6)-]D-Glcp, β-D-Galp-(1 → 3)-[β-D-Galp-(1 → 6)-]D-Glcp and β-D-Galp-(1 → 4)-[β-D-Galp-(1 → 2)-]D-Glcp (Fig. 8a). These eight compounds constitute a class of GOS molecules that are potentially important in promoting the growth of beneficial gut bacteria in a similar manner as HMOs. Due to the limited availability of these GOS molecules at this time, further analysis of the impact of individual molecules is problematic. Attempts to grow *B. thetaiotaomicron* on commercially available 6-galactosyl-lactose as a sole carbon source failed. Perhaps on its own, 6-galactosyl-lactose cannot be processed by *B. thetaiotaomicron* and requires the presence of another compound to initiate metabolism in a sequential manner. The implications of these individual GOS molecules and their function in inducing mucin utilization pathways as single compounds or in combinations warrants further investigation.

Our data provides the first time demonstration that prebiotic GOS mixtures activate the galactan PUL and a subset of mucin glycan utilization PULs in *B. thetaiotaomicron*.

## Discussion

In this study we performed a detailed analysis of the effects of GOS on the activation of PULs in *B. thetaiotaomicron* in order to unravel what features of GOS provide the qualities of hMOS mimicry in intestinal microbiota. We found that consumption of GOS by *B. thetaiotaomicron,* a dominant bacterial species of the distal gastrointestinal tract, results in the activation of a combination of mucin glycan and galactan utilization pathways. Higher DP GOS compounds are metabolized by an endo-galactanase BtGH53, while small DP2 and branched GOS trisaccharides acted as potential inducers for the expression of mucin glycan degradation PUL-encoded systems in *B. thetaiotaomicron*. This was indicated by the presence of SusC and SusD homologs in *B. thetaiotaomicron* culture supernatants which correlated with the bacterium expressing transporters that are involved in the uptake of specific products of glycan degradation^14^,^30^.

We observed that the galactan utilization pathway plays an important role in metabolism of GOS by *B. thetaiotaomicron.* Longer DP GOS carrying β-(1 → 4)-galactan motifs are degraded by the GH53 endo-β-galactanase, whilst some of the mucin-glycan pathway inducer molecules are only uncovered after endo-β-galactanase activity. Therefore bacteria that harbor the ability to degrade galactan will uncover some of the underlying mucin inducer molecules buried in the higher DP GOS molecules. Several members of *Firmicutes* and *Bacteroidetes* encode GH53 endo-β-galactanase enzymes (Table 3)(www.cazy.org/GH53.html)^36^, and interestingly they are found in many *Bifidobacterium* and *Bacteroides* strains that colonize the infant gut. *Bifidobacterium* strains that encode GH53 enzymes include *B. breve, B. longum sub infantis and B. longum sub longum* that are also found in the gut of breast-fed infants^37^ (Table 3). Previous studies on GOS utilization by *Bifidobacterium breve* showed that its endo-β-galactanase played a key role in degrading higher DP GOS^34^. However, it is unknown whether the same mechanism of galactan consumption plus induction of mucin degradation pathways we observed in *B. thetaiotaomicron* is also a feature of GOS utilization in infant gut associated *Bifidobacterium* strains. We can speculate that there may be some similarities because in addition to the presence of GH53 enzymes, *B. breve, B. longum sub infantis and B. longum sub longum* are also considered moderate degraders of mucin^7^,^38^. These specific three strains were also found to be the predominant strains in fecal samples of infants fed GOS/FOS supplemented infant formula and breast-fed infants but not in fecal samples of infants fed standard infant formula^39^. There is evidence to support the theory that GOS promotes the growth of intestinal *Bifidobacterium* strains by inducing both mucin glycan and galactan utilization pathways, however this still needs to be further investigated.

We also demonstrated in our study the ability to correlate different GOS DP fractions with direct physiological effects on the bacterium. Previous studies on GOS consumption by *Bifidobacterium* strains showed that each strain consumed GOS at different rates and growth rate was directly attributed to the ability of the *Bifidobacterium* species to use specific isomers within the GOS mixtures^40^. Our study shows that *B. thetaiotaomicron* uses almost all isomers in the GOS mixture. Using knowledge on what each HPAEC-PAD peak represents in sugar content and linkage type^29^, we can more specifically define, in addition to DP, what GOS isomers are consumed by specific bacteria. Using the more specific definitions of GOS, we can in the future apply this methodology to GOS consumption by other bacterial species.

Based on what is known about the composition of HMOs and core mucin glycans (Fig. 8)^11^,^18^, we hypothesize that the Gal β-(1 → 3)-Glc and 6-GalLac molecules structurally mimic the core mucin and hMOS motifs, while the branched DP3 compound β-D-Galp-(1 → 3)-[β-D-Galp-(1 → 6)-]D-Glcp structurally mimics the branched core mucin glycan motifs found in core 2 and core 4 glycans. Allolactose (Gal-β-(1 → 6)-Glc) present in GOS is typically an inducer of the Lac-operon and is degraded by lactose-specific β-galactosidases^41^. However, since we did not characterize the activation of glycan PULs in *B. thetaiotaomicron* with allolactose as a sole carbon source, we cannot yet exclude it as a potential inducer of mucin glycan associated pathways.

The exact mechanism of structural mimicry of GOS compounds eliciting mucin-glycan utilization systems is still not clear. Whether it is the features of sugar composition or the position of the glycosidic linkage in these particular GOS molecules that activates expression of particular mucin glycan utilization pathways still needs to be investigated. Most core glycan motifs contain GalNac and GlcNac residues that are not found in GOS, suggesting that the linkage type may be more important in this regard than the monosaccharide content. To date the majority of studies concerning bacterial mucin glycan metabolism use oligosaccharides containing N-acetyl-D-lactosamine disaccharide or entire human or pig gastric mucin isolates, which are very complex in size and glycan composition. In this study we identify additional motifs that potentially activate mucin glycan PULs, therefore the knowledge gained in this study is also in identifying specific glycan molecules in GOS that may trigger induction of specific mucin glycan utilization pathways, which to our knowledge has not been observed before.

The presence of specific polysaccharides will dictate which bacteria dominate in their environment^4^,^42^. Galactooligosaccharides are considered a fermentable fibre with potential beneficial health effects by promoting the growth of bacteria in the infant microbiome with a similar composition as in infants that are fed natural mother’s milk^22^. We demonstrate in our current study that the beneficial properties exhibited by GOS as a fermentable fibre by microbial species residing in the gastrointestinal tract are likely two-fold: Firstly, higher DP GOS act as a nutritional source in the form of dietary galactan and the ability to degrade galactan appears to be a characteristic feature of many gut microbial species that are associated with consumption of HMOs. Secondly, GOS mixtures contain molecules that induce mucin glycan utilization pathways which may help promote colonization and adaptation of bacterial species in the GI tract. Mucin glycan foraging is essential for persistence of *B. thetaiotaomicron* in the gut^8^ while HMOs promote intestinal colonization of *B. thetaiotaomicron* via mucin-associated pathways^15^. Therefore the specific GOS molecules identified in this study that trigger mucin glycan utilization pathways may act as “intestinal adaptation factors” to promote colonization and adaptation of mucin-degrading bacteria in the gastrointestinal tract. It is likely that the combination of these effects contributes to the features of GOS as HMO-mimics. The metabolic features of GOS revealed in this study may be useful for the future design of fermentable fibres or prebiotic compounds that selectively promote the growth and colonization of beneficial bacteria, for example by generating new GOS preparations consisting of products of BtGH53-treated pectic galactan combined with DP2 and branched DP3 GOS, or for the selection of probiotic bacterial strains that may be administered in combination with galactooligosaccharides.

## Conclusion

Prebiotic features of GOS is as a combination of galactan plus HMO-like molecules, which stimulated the activation of pectic galactan and mucin-O-glycan degradation pathways in *B. thetaiotaomicron.* Higher DP GOS resembled β-(1 → 4)-galactan, which was shown to be efficiently degraded by an extracellular family GH53 endo-galactanase enzyme. A smaller subset of GOS compounds, namely DP2 and branched DP3 GOS, were attributed to activation of O-mucin glycan degradation pathways, similar to HMOs. These GOS molecules may facilitate colonization of mucus-adapted microbial species in the gastrointestinal tract, therefore future studies into the molecular mechanisms and effects of GOS on bacterial mucin utilization in the gastrointestinal tract is an exciting new frontier for GOS-related research.

## Materials and Methods

### Bacterial Strains, media and reagents

*Bacteroides thetaiotaomicron* VPI-5482 was purchased from DSMZ (DSM 2079, ATCC 29148) (Braunschweig, Germany). The Vivinal ^®^ GOS and TS0903 GOS mixtures were provided by FrieslandCampina (NL). 6-GalactosylLactose was purchased from Carbosynth (UK). Pectic galactan (potato) was purchased from Megazyme (UK). All other media and reagents were purchased from Sigma (Zwijndrecht, Netherlands) unless otherwise stated.

### Isolation of HMOs

HMOs were isolated from a human milk sample that was collected from a volunteer, collecting a full feeding of at least 100 mL around 1 month post-partum. 10 mL human milk samples were centrifuged at 5000 rpm for 30 min at 4 °C. The clear liquid was applied on a graphitized carbon column (10 g, graphitized carbon black, 20–60 mesh, SigmaAldrich) and washed with 30 mL Milli-Q water. The majority of lactose was removed by washing with 30 mL 2% ACN and hMOS were eluted with 40% ACN, containing 0.05% TFA.

### *B. thetaiotaomicron* growth experiments

*B. thetaiotaomicron* growth was carried out as described previously^8^. Briefly, overnight cultures of *B. thetaiotaomicron* were grown at 37 °C in rich medium (Brain Heart infusion broth + 5 mM L-cysteine) under anaerobic conditions in an anaerobic jar (Oxoid) with a GasPak (BD). The following day, 1 ml of a 50 fold dilution containing *B. thetaiotaomicron* overnight culture was prepared in a carbon-limited minimally defined medium of 100 mM KH_2_PO_4_ (pH 7.2), 15 mM NaCl, 8.5 mM (NH_4_)_2_SO_4_, 4 mM L-cysteine, 1.9 mM hematin, 200 mM L-histidine, 100 nM MgCl_2_, 1.4 nM FeSO_4_ · 7 H_2_O, 50 mM CaCl_2_, 1 mg/ml vitamin K3, 5 ng/ml vitamin B12 and individual carbon sources (0.5%, wt/vol). Cultures containing 2 ml total volume were prepared in Hungate tubes under anaerobic conditions using Hungate techniques by flushing sealed culture tubes with 100% CO_2_ gas^43^. Growth curves were obtained by incubating tubes at 37 °C and taking optical density readings at 600 nm (OD600) at ~1–2 h time intervals over a 4 day period. A non-parametric two-tailed Wilcoxon matched pairs signed rank test between glucose and purified GOS (Fig. 2) was carried out using the program GraphPad Prism 7 (Graphpad Software Inc., San Diego, USA).

### HPAEC-PAD

GOS components and products of enzymatic digestion were analyzed by high-pH anion-exchange chromatography on a Dionex DX500 work station equipped with an ED40 pulsed amperometric detection system (HPAEC-PAD) as described previously^26^. The oligosaccharides were separated on a CarboPac PA-1 column (250 by 5 mm; Dionex) using a system of buffer A = 0.1 M NaOH, buffer B = 0.6 M NaOAc in 0.1 M NaOH, buffer C = deionized water and buffer D = 50 mM NaOAc. Separation was performed with 10% A, 85% C and 5% D in 25 min to 40% A, 10% C and 50% D, followed by a 35-min gradient to 75% A, 25% B, directly followed by 5 min washing with 100% B and reconditioning with 10% A, 85% B and 5% D for 7 min.

### Proteomics and enzyme activity analysis

After growth, cultures were centrifuged to obtain supernatants which subsequently were filtered through a 0.2 μm-pore-size syringe filter (Millipore). Identification of proteins in culture supernatants produced by *B. thetaiotaomicron* were analyzed as described previously^30^. For CAZyme activity in culture supernatants, 100 μl of culture supernatant was added to 100 μl of a 10 mg/ml solution of GOS carbohydrate solution in deionized water and incubated at 37 °C overnight to allow the enzyme reactions to proceed. Analysis of the activity of the BtGH53 endo-galactanase on GOS substrates was carried out in Reaction buffer at 37 °C and carbohydrate products were analyzed by HPAEC-PAD (as described above) and by TLC.

Thin-layer chromatography (TLC) of GOS carbohydrates and enzymatic products was completed as described in the literature^44^. Briefly, 2 μl of each reaction mixture was spotted, dried, and then subsequently spotted again on a silica gel 60 plate (Millipore). The solvent system used was 3:1:1 isopropanol, ethylacetate, and deionized water, and plates were run in a TLC jar for approximately 4 to 5 h. Plates were removed from the jar and dried, and spots were visualized by staining with 20% sulfuric acid plus 0.5% orcinol in methanol and heated at 110 °C for half an hour. Plates were scanned and figures prepared using Adobe Photoshop.

### Production of BtGH53

BtGH53 (BT_4668) was amplified from *B. thetaiotaomicron* VPI-5482 genomic DNA by PCR using the Forward primer **CAGGGACCCGGTGAAG**ATGGCCCGGTTACAAATCCTCG and Reverse primer **CGAGGAGAAGCCCGG**TTATTGGATTTTAAAAGCATCTAGTGC containing a stop codon (underlined). The resulting amplified DNA was cloned into a modified pET15b vector (Novagen) containing a LIC (ligation independent cloning) site via the incorporated LIC-specific primer sequences (indicated in bold). The resulting expression vector pBtGH53 encoded BtGH53 with an additional N-terminal His6 tag. Recombinant BtGH53 was produced in *Escherichia coli* BL21*DE3 cells (Novagen) harboring the pBtGH53 plasmid. Cells were grown in Luria Bertani (LB) broth containing ampicillin at a concentration of 100 μg/ml at 37 °C until an OD600 of 1.1 was reached. Protein overproduction was induced by the addition of 1 mM isopropyl β-D-thiogalactopyranoside and cultures were then further incubated for 16 h at 25 °C. Bacterial cells were harvested by centrifugation, resuspended in buffer containing 20 mM Tris pH8.0, 500 mM NaCl, and lysed by sonication. Recombinant His6-tagged BtGH53 was purified from supernatants by immobilized metal affinity chromatography (IMAC) on a nickel-sepharose column (GE Healthcare) preequilibrated with the same buffer and eluted with a gradient of imidazole. Eluted fractions were assessed by SDS-PAGE for the presence of BtGH53 with a size of 39.6 KDa. Pooled fractions of BtGH533 were dialyzed into buffer consisting of 20 mM Tris pH 7.5, 150 mM NaCl (Reaction buffer). The concentration of BtGH53 protein was determined spectrophotometrically at 280 nm using an extinction coefficient of 0.07326 μM^−1^ cm^−1^.

### Determination of BtGH53 specific activities

BtGH53 endo-galactanase activity was determined using potato galactan (Megazyme). A reaction mixture containing 500 μl 2% potato Galactan in 50 mM phosphate buffer pH 7.0 plus 500 μl of 5 μM BtGH53 in the same buffer was incubated for 1 min, 10 min, 60 min and 20 h at 37 °C. After incubation, the reaction was stopped by heating the reactions to 65 degrees celcius for 10 min. The reactions were centrifuged for 10 min at 2800 g. BtGH53 activity on galactan and GOS substrates was analyzed using TLC and HPAEC-PAD as stated above.

## Additional Information

**How to cite this article**: Lammerts van Bueren. A *et al*. Prebiotic galactooligosaccharides activate mucin and pectic galactan utilization pathways in the human gut symbiont *Bacteroides thetaiotaomicron. Sci. Rep.* **7**, 40478; doi: 10.1038/srep40478 (2017).

**Publisher's note:** Springer Nature remains neutral with regard to jurisdictional claims in published maps and institutional affiliations.

## Supplementary Material

Supplementary Information

## Figures and Tables

**Table 1:** Ratios of GOS compounds expressed in a percentage of total GOS mixture as determined by relative peak heights obtained by HPAEC-PAD elution profiles (see Materials and Methods).

GOS component	Percentage (%) of total mixture Vivinal^®^ GOS	TS0903 Purified GOS
***Galactose***	0.68	2.03
***Glucose***	15.34	3.92
**β-D-Galp-(1 → 6)- β -D-Gal**	0	1.32
***Lactose***	19.23	1.08
**β-D-Galp-(1 → 4)- β -D-Gal**	0.95	5.79
**β-D-Galp-(1 → 6)-β-D-Galp-(1 → 4)-D-Glcp (*****6-galactosyl-lactose***)	5.60	3.83
**DP2**^a^	27.25	7.89
**Linear DP3**^b^	14.83	29.19
**Branched DP3**^c^	4.79	6.97
**Linear DP4**^d^	6.60	17.45
**Branched DP4**^d^	1.93	10.14
**≥DP5**	2.80	10.35

^a^lactose derivatives β-D-Galp-(1 → 2)-β-D-Glc, β-D-Galp-(1 → 3)-β-D-Glc and β-D-Galp-(1 → 6)-β-D-Glc.

^b^Includes β-D-Galp-(1 → 4)-β-D-Galp-(1 → 4)-D-Glcp, β-D-Galp-(1 → 4)-β-D-Galp-(1 → 2)-D-Glcp and β-D-Galp-(1 → 4)-β-D-Galp-(1 → 3)-D-Glcp.

^c^Includes β-D-Galp-(1 → 3)-[β-D-Galp-(1 → 6)-]D-Glcp, β-D-Galp-(1 → 2)-[β-D-Galp-(1 → 6)-]D-Glcp and β-D-Galp-(1 → 4)-[β-D-Galp-(1 → 2)-]D-Glcp.

^d^Corresponding DP3 compounds with an additional β-D-Galp-(1 → 4)- at the non-reducing end.

**Table 2:** SusC/D pairs identified in proteomics analysis of *B. thetaiotaomicron* culture supernatants after growth on lactose, galactooligosaccharides and hMOS.

	Lactose	GOS	hMOS	hMOS (a)
**SusC gene ID**				
BT_0317	−	−	+	−
BT_0439	−	+	+	−
BT_0867	−	−	+	−
BT_1040	−	−	+	+
BT_1631	−	−	+	−
BT_2032	+	+	+	−
BT_2626	−	−	−	+
BT_2805	−	+	+	+
BT_3958	−	+	+	+
BT_4039	−	−	+	+
BT_4135	−	−	+	+
BT_4247	−	+	+	−
BT_4298	−	+	+	+
BT_4671	−	+	−	−
**SusD gene ID**				
BT_0318	−	−	+	−
BT_0866	−	−	+	−
BT_1039	−	+	+	+
BT_1630	−	−	+	−
BT_2033	+	+	+	−
BT_2365	−	+	+	−
BT_2559	−	+	+	−
BT_2625	−	+	+	+
BT_2806	−	−	+	+
BT_3520	−	+	+	−
BT_3959	−	+	+	+
BT_3984	−	−	+	−
BT_4038	−	−	+	+
BT_4134	−	−	+	+
BT_4246	−	+	+	−
BT_4297	−	+	+	+
BT_4670	−	+	−	−

Proteins identified are unique PULs after considering glucose controls. A full list of proteins identified can be found in the accompanying Supplementary Material.

^a^As identified in ref. 15.

**Table 3:** The presence of GH53 enzymes in microbial strains associated with gut microbiota.

**Probiotic bacteria**	*Bifidobacterium breve*
	*Bifidobacterium dentium*
	*Bifidobacterium longum*
	*Bifidobacterium longum sub. infantis*
	*Bifidobacterium longum sub. longum*
	*Lactobacillus reuteri*
**Human gut commensal bacteria**	*Bacteroides dorei*
	*Bacteroides thetaiotaomicron*
	*Enterococcuis faecium*
	*Eubacterium rectale*
	*Prevotella dentalis*
	*Prevotella denticola*
	*Prevotella ruminicola*
	*Ruminococcus albus*

## References

[b1] Hooper L. V. & Macpherson A. J. Immune adaptations that maintain homeostasis with the intestinal microbiota. Nat. Rev. Immunol. 10, 159–69 (2010). 20182457 10.1038/nri2710

[b2] Notghi A., Hutchinson R., Kumar D., Smith N. B. & Harding L. K. Simplified method for the measurement of segmental colonic transit time. Gut 35, 976–981 (1994). 8063227 10.1136/gut.35.7.976 PMC1374847

[b3] David L. a. . Diet rapidly and reproducibly alters the human gut microbiome. Nature 505, 559–63 (2014). 24336217 10.1038/nature12820 PMC3957428

[b4] Rogowski A. . Glycan complexity dictates microbial resource allocation in the large intestine. Nat. Commun. 6, 7481, doi: 10.1038/ncomms8481 (2015). 26112186 PMC4491172

[b5] Martens E. C. . Recognition and Degradation of Plant Cell Wall Polysaccharides by Two Human Gut Symbionts. PLoS Biol. 9, e1001221 (2011). 22205877 10.1371/journal.pbio.1001221 PMC3243724

[b6] Milani C. . Bifidobacteria exhibit social behavior through carbohydrate resource sharing in the gut. Sci. Rep. 5, doi: 10.1038/srep15782 (2015). PMC4623478 26506949

[b7] Tailford L. E., Crost E. H., Kavanaugh D. & Juge N. Mucin glycan foraging in the human gut microbiome. Front. Genet. 6, 81, doi: 10.3389/fgene.2015.00081 (2015). 25852737 PMC4365749

[b8] Martens E. C., Chiang H. C. & Gordon J. I. Mucosal Glycan Foraging Enhances Fitness and Transmission of a Saccharolytic Human Gut Bacterial Symbiont. Cell Host Microbe 4, 447–457 (2008). 18996345 10.1016/j.chom.2008.09.007 PMC2605320

[b9] Li H. . The outer mucus layer hosts a distinct intestinal microbial niche. Nat. Commun, doi: 10.1038/ncomms9292 (2015). PMC4595636 26392213

[b10] Corfield A. P. Mucins: A biologically relevant glycan barrier in mucosal protection. Biochim. Biophys. Acta - Gen. Subj. 1850, 236–252 (2015). 10.1016/j.bbagen.2014.05.003 24821013

[b11] Bergstrom K. S. B. & Xia L. Mucin-type O-glycans and their roles in intestinal homeostasis. Glycobiology 23, 1026–1037 (2013). 23752712 10.1093/glycob/cwt045 PMC3858029

[b12] Robbe C., Capon C., Coddeville B. & Michalski J.-C. Structural diversity and specific distribution of O-glycans in normal human mucins along the intestinal tract. Biochem. J. 384, 307–16 (2004). 15361072 10.1042/BJ20040605 PMC1134114

[b13] Sonnenburg J. L. . Glycan foraging *in vivo* by an intestine-adapted bacterial symbiont. Science 307, 1955–9 (2005). 15790854 10.1126/science.1109051

[b14] Martens E. C., Koropatkin N. M., Smith T. J. & Gordon J. I. Complex glycan catabolism by the human gut microbiota: the Bacteroidetes Sus-like paradigm. J. Biol. Chem. 284, 24673–7 (2009). 19553672 10.1074/jbc.R109.022848 PMC2757170

[b15] Marcobal A. . Bacteroides in the infant gut consume milk oligosaccharides via mucus-utilization pathways. Cell Host Microbe 10, 507–14 (2011). 22036470 10.1016/j.chom.2011.10.007 PMC3227561

[b16] Bode L. Human milk oligosaccharides: every baby needs a sugar mama. Glycobiology 22, 1147–62 (2012). 22513036 10.1093/glycob/cws074 PMC3406618

[b17] Bode L. The functional biology of human milk oligosaccharides. Early Hum. Dev. 1–4, doi: 10.1016/j.earlhumdev.2015.09.001 (2015). 26375354

[b18] Marcobal a. & Sonnenburg J. L. Human milk oligosaccharide consumption by intestinal microbiota. Clin. Microbiol. Infect. 18 Suppl 4, 12–5 (2012). 22647041 10.1111/j.1469-0691.2012.03863.x PMC3671919

[b19] Newburg D. S., Ruiz-Palacios G. M. & Morrow A. L. Human Milk Glycans Protect Infants Against Enteric Pathogens. Annu. Rev. Nutr. 25, 37–58 (2005). 16011458 10.1146/annurev.nutr.25.050304.092553

[b20] Manthey C. F., Autran C. a., Eckmann L. & Bode L. Human Milk Oligosaccharides Protect Against Enteropathogenic Escherichia coli Attachment *In Vitro* and EPEC Colonization in Suckling Mice. J. Pediatr. Gastroenterol. Nutr. 58, 167–170 (2014). 10.1097/MPG.0000000000000172 PMC8865036 24048169

[b21] Ben X. . Supplementation of milk formula with galacto-oligosaccharides improves intestinal micro-flora and fermentation in term infants. Chin. Med. J. (Engl). 117, 927–931 (2004). 15198901

[b22] Davis L. M. G., Martínez I., Walter J., Goin C. & Hutkins R. W. Barcoded pyrosequencing reveals that consumption of galactooligosaccharides results in a highly specific bifidogenic response in humans. PLoS One 6, e25200 (2011). 21966454 10.1371/journal.pone.0025200 PMC3180383

[b23] Garrido D. . Utilization of galactooligosaccharides by Bifidobacterium longum subsp. infantis isolates. Food Microbiol. 33, 262–270 (2013). 23200660 10.1016/j.fm.2012.10.003 PMC3593662

[b24] Prenosil J. E., Stuker E. & Bourne J. R. Formation of oligosaccharides during enzymatic lactose: Part I: State of art. Biotechnol. Bioeng. 30, 1019–1025 (1987). 18581545 10.1002/bit.260300904

[b25] van Leeuwen S. S., Kuipers B. J. H., Dijkhuizen L. & Kamerling J. P. Comparative structural characterization of 7 commercial galacto-oligosaccharide (GOS) products. Carbohydr. Res. 425, 48–58 (2016). 27035911 10.1016/j.carres.2016.03.006

[b26] van Leeuwen S. S., Kuipers B. J. H., Dijkhuizen L. & Kamerling J. P. 1H NMR analysis of the lactose/β-galactosidase-derived galacto-oligosaccharide components of Vivinal^®^ GOS up to DP5. Carbohydr. Res. 400, 59–73 (2014). 25444767 10.1016/j.carres.2014.08.012

[b27] Park A.-R. & Oh D.-K. Galacto-oligosaccharide production using microbial β-galactosidase: current state and perspectives. Appl. Microbiol. Biotechnol. 85, 1279–1286 (2010). 19943044 10.1007/s00253-009-2356-2

[b28] Gietl E. . Factors Involved in the *In Vitro* Fermentability of Short Carbohydrates in Static Faecal Batch Cultures. Int. J. Carbohydr. Chem. 2012, 1–10 (2012).

[b29] van Leeuwen S. S., Kuipers B. J. H., Dijkhuizen L. & Kamerling J. P. Development of a (1)H NMR structural-reporter-group concept for the analysis of prebiotic galacto-oligosaccharides of the [β-d-Galp-(1 → x)]n-d-Glcp type. Carbohydr. Res. 9–13, doi: 10.1016/j.carres.2014.08.011 (2014). 25249391

[b30] Lammerts van Bueren A., Saraf A., Martens E. C. & Dijkhuizen L. Differential Metabolism of Exopolysaccharides from Probiotic Lactobacilli by the Human Gut Symbiont Bacteroides thetaiotaomicron. Appl. Environ. Microbiol. 81, 3973–3983 (2015). 25841008 10.1128/AEM.00149-15 PMC4524142

[b31] Cho K. H., Cho D., Wang G. R. & Salyers A. A. New regulatory gene that contributes to control of Bacteroides thetaiotaomicron starch utilization genes. J. Bacteriol. 183, 7198–7205 (2001). 11717279 10.1128/JB.183.24.7198-7205.2001 PMC95569

[b32] Martens E. C., Koropatkin N. M., Smith T. J. & Gordon J. I. Complex Glycan Catabolism by the Human Gut Microbiota: The Bacteroidetes Sus-like Paradigm. J. Biol. Chem. 284, 24673–24677 (2009). 19553672 10.1074/jbc.R109.022848 PMC2757170

[b33] Ryttersgaard C. . The structure of endo-beta-1,4-galactanase from Bacillus licheniformis in complex with two oligosaccharide products. J. Mol. Biol. 341, 107–17 (2004). 15312766 10.1016/j.jmb.2004.05.017

[b34] O’Connell Motherway M., Kinsella M., Fitzgerald G. F. & van Sinderen D. Transcriptional and functional characterization of genetic elements involved in galacto-oligosaccharide utilization by *Bifidobacterium breve* UCC2003. Microb. Biotechnol. 6, 67–79 (2013). 23199239 10.1111/1751-7915.12011 PMC3815386

[b35] Torpenholt S. . Activity of three β−1,4-galactanases on small chromogenic substrates. Carbohydr. Res. 346, 2028–2033 (2011). 21696710 10.1016/j.carres.2011.05.017

[b36] Lombard V., Golaconda Ramulu H., Drula E., Coutinho P. M. & Henrissat B. The carbohydrate-active enzymes database (CAZy) in 2013. Nucleic Acids Res. 42, D490–D495 (2014). 24270786 10.1093/nar/gkt1178 PMC3965031

[b37] Sela D. a. Bifidobacterial utilization of human milk oligosaccharides. Int. J. Food Microbiol. 149, 58–64 (2011). 21342711 10.1016/j.ijfoodmicro.2011.01.025

[b38] Ruas-Madiedo P., Gueimonde M., Fernandez-Garcia M., de los Reyes-Gavilan C. G. & Margolles a. Mucin Degradation by Bifidobacterium Strains Isolated from the Human Intestinal Microbiota. Appl. Environ. Microbiol. 74, 1936–1940 (2008). 18223105 10.1128/AEM.02509-07 PMC2268317

[b39] Haarman M. & Knol J. Quantitative Real-Time PCR Assays To Identify and Quantify Fecal Bifidobacterium Species in Infants Receiving a Prebiotic Infant Formula Quantitative Real-Time PCR Assays To Identify and Quantify Fecal Bifidobacterium Species in Infants Receiving a Prebio. Appl. Environ. Microbiol. 71, 2318–2324 (2005). 15870317 10.1128/AEM.71.5.2318-2324.2005 PMC1087546

[b40] Peacock K. S., Ruhaak L. R., Tsui M. K., Mills D. a. & Lebrilla C. B. Isomer-specific consumption of galactooligosaccharides by bifidobacterial species. J. Agric. Food Chem. 61, 12612–9 (2013). 24313277 10.1021/jf403789r PMC3912189

[b41] Wheatley R. W., Lo S., Jancewicz L. J., Dugdale M. L. & Huber R. E. Structural Explanation for Allolactose (lac Operon Inducer) Synthesis by lacZ -Galactosidase and the Evolutionary Relationship between Allolactose Synthesis and the lac Repressor. J. Biol. Chem. 288, 12993–13005 (2013). 23486479 10.1074/jbc.M113.455436 PMC3642343

[b42] Koropatkin N. M., Cameron E. a. & Martens E. C. How glycan metabolism shapes the human gut microbiota. Nat. Rev. Microbiol. 10, 323–35 (2012). 22491358 10.1038/nrmicro2746 PMC4005082

[b43] Hungate R. E. The Anaerobic Mesophilic Cellulolytic Bacteria. Microbiol. Mol. 14, 1–49 (1950). 10.1128/br.14.1.1-49.1950 PMC440953 15420122

[b44] Koropatkin N. M. & Smith T. J. SusG: a unique cell-membrane-associated alpha-amylase from a prominent human gut symbiont targets complex starch molecules. Structure 18, 200–215 (2010). 20159465 10.1016/j.str.2009.12.010

