# Supplementary material for: Prebiotic galactooligosaccharides activate mucin and pectic galactan utilization pathways in the human gut symbiont Bacteroides thetaiotaomicron
Source: Sci Rep. 2017 Jan 16;7:40478. doi: 10.1038/srep40478 (PMC5238430; doi:10.1038/srep40478)
Supplement: Supplementary Information [file srep40478-s1.pdf]

Supplementary Material: **Prebiotic galactooligosaccharides activate mucin and pectic galactan utilization pathways in the human gut symbiont Bacteroides thetaiotaomicron**

Alicia Lammerts van Bueren[1*], Marieke Mulder, Sander van Leeuwen, Lubbert Dijkhuizen[1]

Supplementary Figure 1: NMR analysis of compound remaining after *B. thetaiotaomicron* growth on purified GOS mixture. Compound was isolated from culture supernatants of culture filtrate and analyzed using previously reported H^1 NMR methods (1) and verified to be β-D-Galp-(1-4)-[β-D-Galp-(1-6)-]D-Glcp.

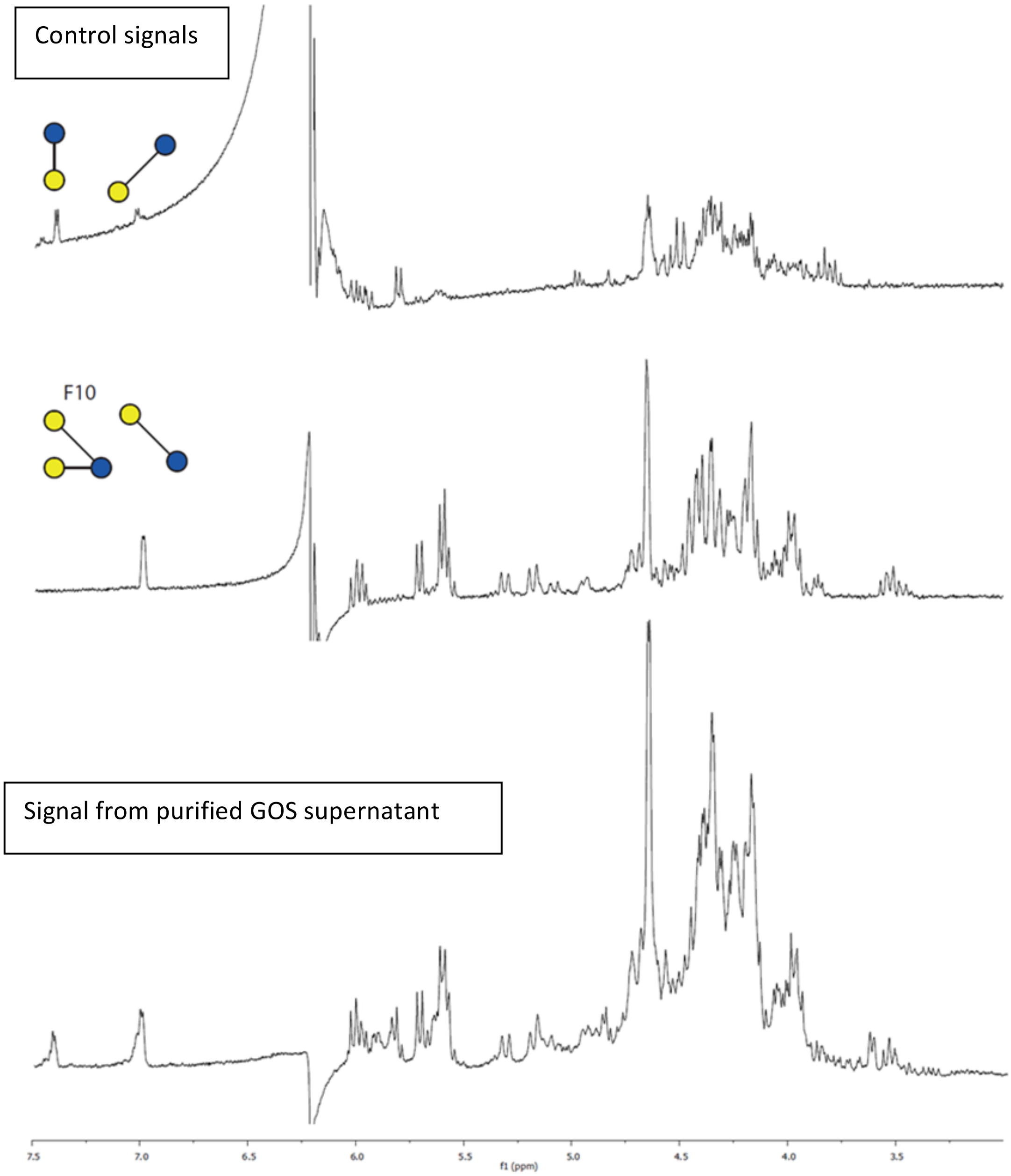

Supplementary Figure 2: HMO Growth curves of B. thetaiotaomicron grown in minimally defined medium with carbon sources added at a final concentration of 5 mg/ml. hMOS (triangles), lactose (squares), glucose (diamonds) and no carbon (NC) control (circles). Cultures were grown in Hungate tubes flushed with 100% CO2. All experiments are the average of three experiments and standard deviations are indicated in black.

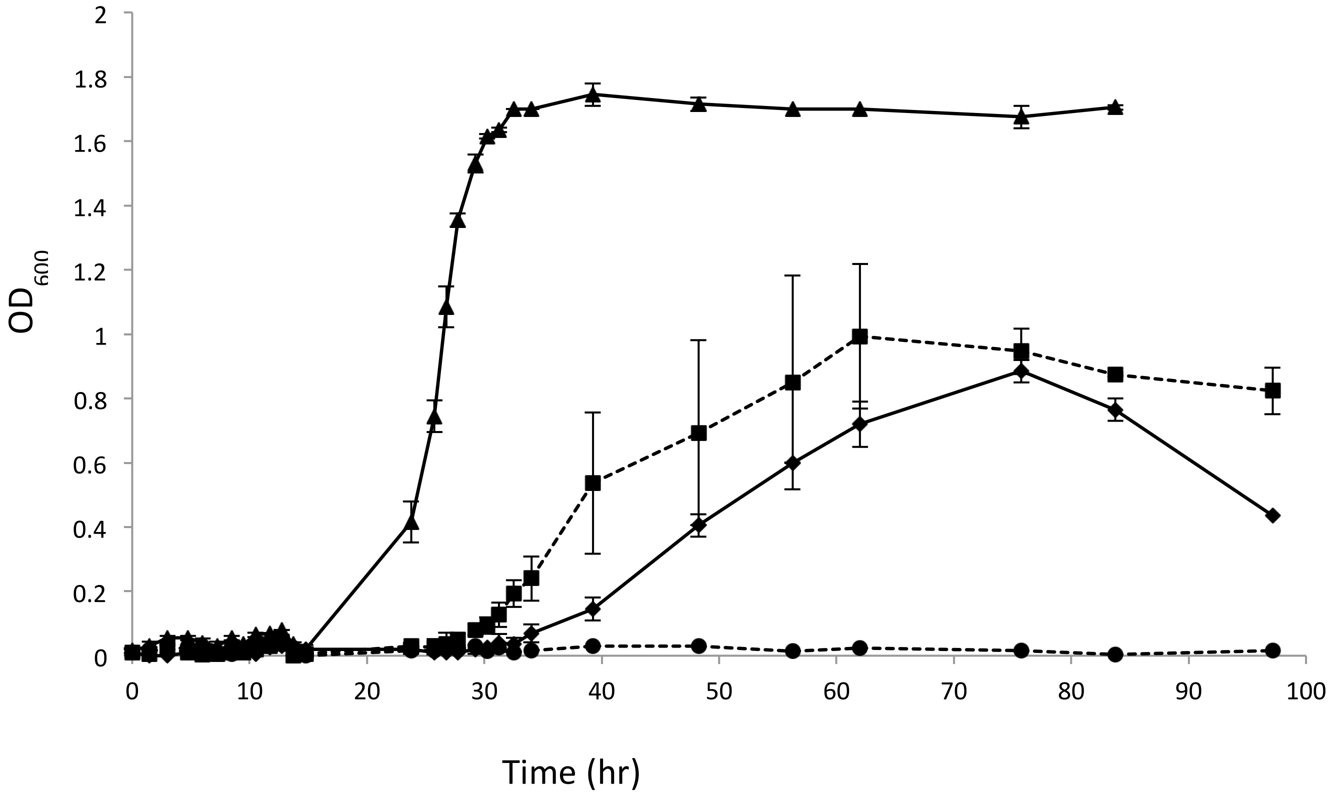

Reference

(1) Leeuwen, S.S. van, Kuipers, B.J.H., Dijkhuizen, L., and Kamerling, J.P. (2014) Development of a (1)H NMR structural-reporter-group concept for the analysis of prebiotic galacto-oligosaccharides of the [β-d-Galp-(1→x)]n-d-Glcp type. *Carbohydr Res* 9–13
